# Measuring DNA hybridization using fluorescent DNA-stabilized silver clusters to investigate mismatch effects on therapeutic oligonucleotides

**DOI:** 10.1186/s12951-018-0361-2

**Published:** 2018-04-06

**Authors:** Donny de Bruin, Nelli Bossert, Annemieke Aartsma-Rus, Dirk Bouwmeester

**Affiliations:** 1 Leiden Institute of Physics, Leiden University, Leiden, 2333 CA The Netherlands; 2 Leiden University Medical Center, Leiden, 2333 ZA The Netherlands; 3 Department of Physics, University of California, Santa Barbara, CA 93106 USA

**Keywords:** DNA hybridization, Mismatch dependencies, Therapeutic oligonucleotides, Silver nanoparticles

## Abstract

**Background:**

Short nucleic acid oligomers have found a wide range of applications in experimental physics, biology and medicine, and show potential for the treatment of acquired and genetic diseases. These applications rely heavily on the predictability of hybridization through Watson–Crick base pairing to allow positioning on a nanometer scale, as well as binding to the target transcripts, but also off-target binding to transcripts with partial homology. These effects are of particular importance in the development of therapeutic oligonucleotides, where off-target effects caused by the binding of mismatched sequences need to be avoided.

**Results:**

We employ a novel method of probing DNA hybridization using optically active DNA-stabilized silver clusters (Ag-DNA) to measure binding efficiencies through a change in fluorescence intensity. In this way we can determine their location-specific sensitivity to individual mismatches in the sequence. The results reveal a strong dependence of the hybridization on the location of the mismatch, whereby mismatches close to the edges and center show a relatively minor impact. In parallel, we propose a simple model for calculating the annealing ratios of mismatched DNA sequences, which supports our experimental results.

**Conclusion:**

The primary result shown in this work is a demonstration of a novel technique to measure DNA hybridization using fluorescent Ag-DNA. With this technique, we investigated the effect of mismatches on the hybridization efficiency, and found a significant dependence on the location of individual mismatches. These effects are strongly influenced by the length of the used oligonucleotides. The novel probe method based on fluorescent Ag-DNA functions as a reliable tool in measuring this behavior. As a secondary result, we formulated a simple model that is consistent with the experimental data.

**Electronic supplementary material:**

The online version of this article (10.1186/s12951-018-0361-2) contains supplementary material, which is available to authorized users.

## Background

Nucleic acid oligomers have an increasingly large number of applications in modern science. Their common availability and the reliability of specific Watson–Crick base pairing has allowed short, modified DNA sequences to be used as spacers and positioning tools [1–3] on a nanometer scale. In molecular biology, various probing systems can be constructed in the form of DNA microarrays, in order to measure processes like gene expression in bulk [4]. Another important application of oligonucleotides is the development of therapies. There are several ways oligonucleotides can be exploited for this, with single stranded antisense oligonucleotides (AONs) for splicing modulation, recently gaining attention [5–7]. In 2016, two AONs, eteplirsen and nusinersen, were approved by FDA, for the treatment of Duchenne muscular dystrophy and spinal muscular atrophy, respectively [8]. For the development of well-functioning AONs, such as these for application in patients, a good understanding of the hybridization to the target RNA is essential.

While the energetics of DNA hybridization are relatively well understood [9–12], precise experiments require potentially aggressive modifications such as the addition of fluorescent dyes [13]. These modifications can affect DNA conformation and the stability of the double helix [14, 15], and are usually performed on surface-bound DNA in microarrays, rather than in cells or free solution. The different environments and the unpredictable effect of modifications make it uncertain whether or not previous experiments represent the behavior in cells accurately. For AON therapeutics, it is of particular importance to understand how nucleotide mismatches in the sequence affect their effectiveness, to ensure specific binding to the target sequence while avoiding interfering with other, partially homologous, sequences.

Combining the virtue of DNA as a nanoscale positioning tool, and the optical properties of noble metal nanoparticles, has led to the construction of a wide array of signaling tools for use in biology and medicine [16, 17]. These applications include fluorescent molecular beacons, for signaling drug delivery [18], gene specific silencing [19], and mRNA detection and regulation [20, 21]. In this work we introduce a novel experimental method, based on the exceptional optical properties of fluorescent DNA-stabilized silver clusters (Ag-DNA, for a comprehensive review see [22]), for studying the binding efficiencies of short DNA sequences. Fluorescent Ag-DNA is synthesized on short sections of single stranded DNA (ssDNA), and the optical properties exhibit a strong dependency on the DNA sequence and length [23–26]. Variations in the DNA scaffold lead to fluorescence ranging throughout the visible range, with varying quantum yields, resulting in a characteristic fluorescence signal strongly specific to the used ssDNA sequence. We utilize these properties to develop a method to express DNA hybridization through a distinct change in Ag-DNA fluorescence intensity. We employ a 19-base DNA sequence as a probe (19b-Probe, based on [27]). This probe has been shown to yield a strong fluorescence emission around 562 nm after silver is added and chemically reduced. The 19b-Probe is attached to sequences which are complementary to parts of exon 51 in the dystrophin transcript. An out-of-frame mutation of the dystrophin pre-mRNA leads to the development of Duchenne muscular dystrophy (DMD) [5]. Using an AON that can hybridize with a target exon in the dystrophin pre-mRNA splicing can be modulated such that the reading frame is restored and a partial functional dystrophin can be produced. These sequences can therefore potentially be implemented in RNA form as AONs in the treatment of Duchenne muscular dystrophy, modulating splicing by hybridizing with the pre-mRNA [5]. The binding efficiency, and therefore the effectiveness of these AONs, can be determined by measuring the Ag-DNA fluorescence. Because the probe consists of DNA, the only modification to the AON prior to hybridization is the addition of this DNA fragment. The effectiveness of DNA–DNA binding is then expressed through the fluorescence intensity of the 19b-Probe, when it is used to stabilize a silver cluster. This allows us to study the hybridization efficiency in equilibrium without adding foreign objects such as fluorescent dyes.

We apply this simple and non-invasive method to study the effect of nucleotide mismatches on DNA hybridization. This topic is important for medical AON applications, as they rely heavily on the predictability of target-specific binding. In this article, we demonstrate a strong location dependency for mismatched DNA–DNA binding rates. Furthermore, we introduce a simple theoretical model to explain the experimental results.

## Methods

DNA strands were purchased from Integrated DNA Technologies with standard desalting, and used without further purification. All DNA sequences are made available as Additional file 1: Table S1. Strands were prepared and mixed in 25 mM HEPES–NaOH, pH 7.4, and mixed strands were allowed to hybridize for 2 h at 37 °C. Silver cluster synthesis was performed through addition of AgNO_3_ (99.9999%, Sigma Aldrich) in a 9.6:1 AgNO_3_:Probe ratio, followed by reduction with NaBH_4_ (99%, Sigma Aldrich) in a 4.6:1 NaBH_4_:Probe ratio, after a 30 min incubation. Samples were allowed to stabilize their fluorescence for 60 min after reduction. The fluorescence was monitored continuously during this time to ensure stability before proceeding. Measurements were then performed by collecting emission spectra on a Cary Eclipse fluorimeter (Varian), fitted with a Peltier element and controller to maintain the temperature of 37 °C. All measurements were performed three times under identical conditions. The standard deviation between measurements is presented as error bars in the graphs. Data processing and application of the model made use of the Matlab2012a software suite (The MathWorks, Inc).

## Results

### Ag-DNA probe concept

Our technique involves the expression of Ag-DNA fluorescence to represent DNA-hybridization. To facilitate this, the AON sequence of interest is modified on the 5′-end by the addition of the 19b-Probe (Probe-AON sequence). The AON used is complimentary to a 90-nucleotide part of an exon of the dystrophin transcript, which is used as the binding target (Target sequence).

**Fig. 1:**
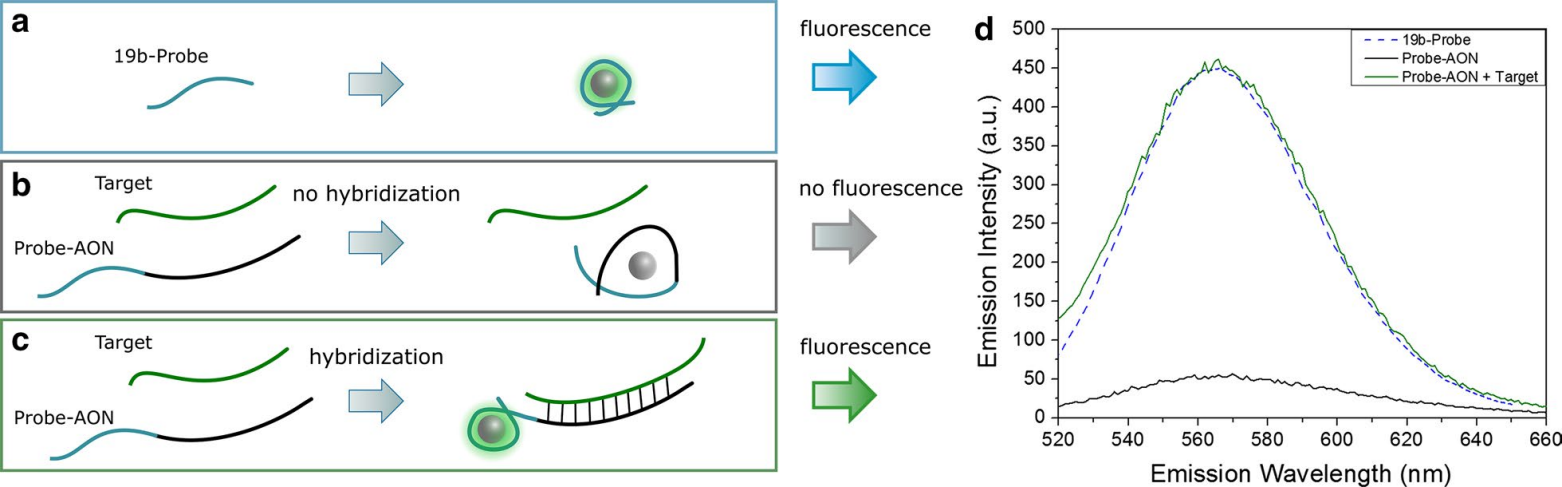
Cartoons of the probe concept (left), and the measured fluorescence spectra (right). The 19b-Probe sequence (**a**) produces a strong fluorescence centered on 562 nm emission (**d**, blue, dashed curve), after silver is added and reduced. The unbound Probe-AON sequence (**b**) does not yield a significant fluorescence (**d**, black curve) when it is not bound to the target strand. When the Probe-AON sequence binds to the Target sequence (**c**), a strong fluorescent distribution can be produced which is comparable to that of the 19b-Probe (**d**, green curve). Experiments were performed with DNA concentrations of 20 µM, mixing the two sequences in a 1:1 ratio

The 19b-Probe (Fig. 1a), unbound and in free solution, shows a strong fluorescence after addition and reduction of silver ions, in agreement with previous studies (Fig. 1d, blue curve) [27]. However, when the probe is appended to an AON-sequence (Fig. 1b), this fluorescence is not significantly expressed (Fig. 1d, black curve), as it heavily depends on the size and shape of the stabilized silver cluster and its DNA environment [22]. The now much longer DNA sequence is expected to produce different, typically larger, Ag-DNA constructs, which strongly suppresses the characteristic fluorescence around 562 nm (Fig. 1d, black curve).

However, when the Probe-AON sequence fully hybridizes with the Target sequence (Fig. 1c), the 19b-Probe sequence remains as an ssDNA appendix to the double-stranded AON-Target. The rigid double stranded AON-Target construct can now be viewed as a handle that holds the 19b-Probe at one end. The probe is then expected to represent a similar DNA environment to the silver clusters as when it is unbound, resulting in the distinct fluorescence centered around 562 nm. The silver ions are not expected to interact strongly with the bound DNA–DNA hybrids [3], and we have confirmed experimentally that the AON-Target constructs do not produce fluorescence. When the Probe-AON and Target sequences are mixed in bulk and allowed to hybridize, we observed a large amount of fluorescent silver clusters after silver addition (Fig. 1d, green curve). The 562 nm peak and the Gaussian shape of the spectrum are consistent with the fluorescence exhibited by the 19b-Probe.

The strength of the emission intensity represents the number of fluorescent clusters that form, allowing us to treat it as a measure of the number of hybridized strands. DNA–DNA hybridization, cluster synthesis and measurements are performed at 37 °C, in HEPES–NaOH pH 7.4 to approach physiological conditions. Additionally, the use of the 90-base Target sequence, which is significantly longer than the 23-nucleotide AON, allows us to more closely mimic the conditions in living cells, where the target RNA is generally much longer than the AON.

### Measured nucleotide-mismatch dependencies

We utilize our method of detecting DNA–DNA binding to analyze the nucleotide-mismatch dependency on DNA hybridization. This is facilitated by introducing individual mismatches in various locations to the AON sequence attached to the 19b-Probe. We performed a series of measurements that included single mismatches on each possible location in the used AON. The effectiveness of hybridization to the target sequence is determined by measuring the fluorescence intensity. The experiments are performed on two different AONs, AON1 and AON2, 23 and 20 bases in length respectively. Both AONs are complimentary to a different part of exon 51 of the dystrophin transcript. A thymine-base was used to replace one nucleotide in the AON-Probe sequence for all mismatch locations, unless a thymine was already present, in which case an adenine was used (Additional file 1: Table S1).

**Fig. 2:**
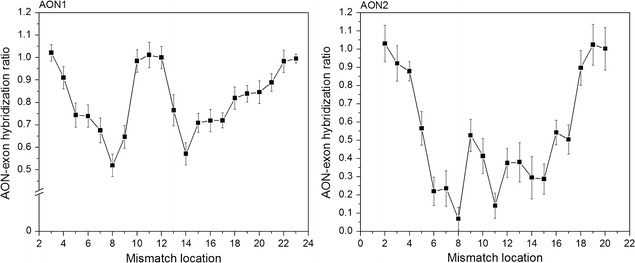
Measured DNA hybridization ratios for the AON1 (left) and AON2 (right) sequences, with various mismatch locations. The total fluorescence intensity is measured by integrating the emission spectra, as measured by fluorimeter using a 495 nm excitation. The data is normalized with respect to a sample without mismatches, which represents 1.0 on the y-axis. Error bars represent the standard deviation measured through experimental repetition (N = 4)

Our main experimental results are shown in Fig. 2. Samples are produced for every mismatch location, and emission spectra are collected between 510 and 650 nm by use of a fluorimeter, using a 495 nm excitation. The number of AONs bound to the target sequence is represented by the total fluorescence intensity collected from the samples, as can be determined by integrating the spectra over all wavelengths. However, because the fluorescence spectrum of the 19b-Probe is Gaussian, it exhibits a symmetry in the emission peak, allowing us to use an integral of either half of the spectrum to determine the fluorescence intensity. Hence, we limited the integral to emission wavelengths greater than the emission peak, 562 nm, to minimize the effect of scattering from the excitation. A sample without mismatches is used to normalize the data, by representing a hybridization ratio of 1.0.

In our experiments, we investigated how DNA–DNA binding is affected when mismatches are present in the sequence. In literature, it can be found that the binding is more sensitive to mismatches closer to the middle of the sequence [13, 28, 29], leading us to expect a single, roughly symmetrical dip in the hybridization ratio. However, in our experiments, we observe sharp peaks of increased binding when the mismatch is close to the center of the sequence, in particular for AON1. These results are in line with more recent work involving surface-bound DNA in microarrays [29], where complex behavior similar to this has been suggested.

### Theoretical predictions of nucleotide-mismatch dependencies

To support our measured results, we propose a simple model to calculate binding efficiencies between mismatched DNA sequences.

In general, the expected binding rates of two DNA strands can be calculated explicitly from the sequences in the nearest-neighbor model. Within this model, interactions between sequential DNA bases are taken into account, and the results are relatively well confirmed by experimental melting curves [9, 10].

When including mismatches, the still commonly used double-ended zipper model [30, 31] assumes that DNA can only separate, or ‘unzip’, from the ends, leading it to predict the binding rates to be independent on mismatch location. However, our measured behavior, as well as other recent results [13, 29] suggest that this may not be the case, leading us to suggest a new way of calculating this behavior.

The changes in enthalpy (ΔH) and entropy (ΔS) upon the binding of two DNA strands of length N can be determined from

1 $$\Delta H = \Delta h_{0} + \sum\limits_{i = 1}^{N} {\Delta h_{i}} \quad \Delta S = \Delta s_{0} + \sum\limits_{i = 1}^{N} {\Delta S_{i}}$$

using the previously determined energy and entropy changes from an individual basepair (Δh_i_, Δs_i_) in the nearest neighbor model [9, 10] Δh_0_ and Δs_0_ represent initiation terms that are determined by whether the first base pair of the sequence is an A-T or G-C. The melting temperature, T_m_, can then be determined from [32, 33]

2 $$T_{m} = \frac{\Delta H}{\Delta S + R\ln \left(C/4\right)} + 16.6\log [Na^{+}]$$

where R is the universal gas constant, C is the total concentration of oligonucleotides when both strands are in equal numbers, and [Na^+^] the molar concentration of monovalent cations in the solution.

In our experiments, we measure the total fluorescence intensity coming from our samples, which represents the number of fluorescent silver clusters stabilized by the 19b-Probe. Because the fluorescence is strongly sequence-specific, the measured fluorescence centered on 562 nm is specifically produced when the 19-base sequence is isolated as single stranded DNA. The fluorescence intensity therefore represents the number of Probe-AON sequences bound to the target sequence, from which the fraction of annealed hybrids (θ) in thermodynamic equilibrium is determined. From the energies and melting temperature, we can derive this number from the melting curves of bimolecular reactions [34]

3 $$\theta = 1 - \frac{2}{{1 + \sqrt {1 + 8e^{- \chi}}}},\,{\text{with}} \quad \chi = \frac{\Delta H}{R}\left({\frac{1}{T} - \frac{1}{{T_{m}}}} \right)$$

where the temperature (T) is kept at 310 K in our experiments.

To account for individual mismatches, we start by treating both halves of the DNA strands on either side of the mismatch as individual pairs, and calculate the energy and entropy changes, ΔH_left/right_ and ΔS_left/right_ individually. The expected binding ratios for the two halves, θ_left/right_, can then be derived using Eq. 3. We model the interaction between the two halves in the form of a coupling term:

4 $$\Delta H^{\prime}_{left/right} = \Delta H_{left/right} + J_{H} (1 - \theta_{right/left}),\,{\text{and}}$$

5 $$\Delta S^{\prime}_{left/right} = \Delta S_{left/right} + J_{S} (1 - \theta_{right/left})$$

where the coupling factors J_H_, J_S_ are positive free parameters which account for the fact that the two halves on either side of the mismatch are still connected. The coupling factors are positive corrections to the (negative) changes in energy, and are used to approximate the destabilizing effect of the mismatch. In particular, they represent the mechanical effect of the extra mass of the other half ‘pulling’ on the sequence if it is unbound, represented by the probability (1 − θ_right/left_), as it moves around in Brownian motion. If the other half is bound, we expect any stabilizing effect on the binding to be negligible due to the high flexibility of single stranded DNA. The average change in energy per pair of DNA strands is then calculated by weighting these energies with their (coupling-corrected) binding probabilities, θ′_right/left_, again determined using Eq. 3:

6 $$\overline{{\Delta {\rm H}}} = \Delta H^{\prime}_{left} \theta^{\prime}_{left} + \Delta H^{\prime}_{right} \theta^{\prime}_{right}$$

7 $$\overline{\Delta S} = \Delta S^{\prime}_{left} \theta^{\prime}_{left} + \Delta S^{\prime}_{right} \theta^{\prime}_{right}$$

These energies are then used to calculate the expected ratio of hybridized DNA strands using Eq. 3, to be compared with our experimental data.

**Fig. 3:**
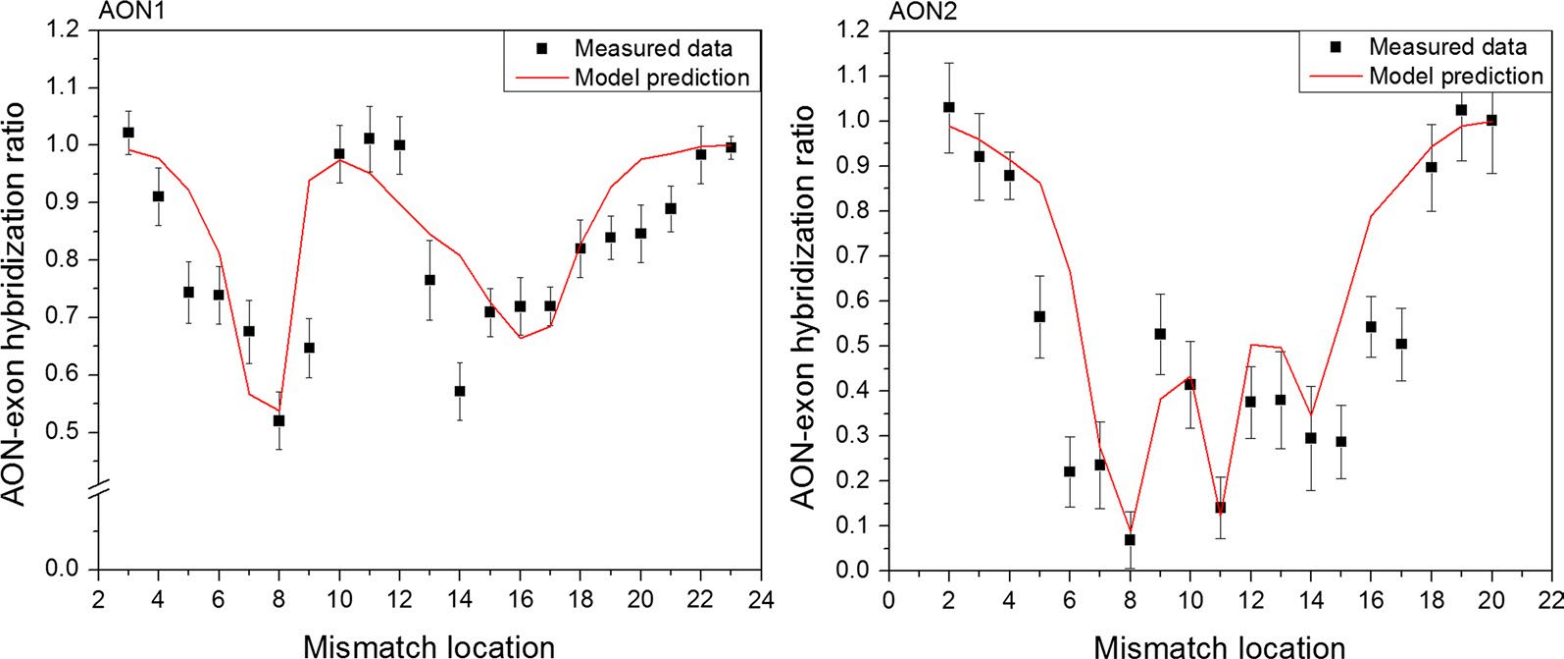
Measured DNA–DNA hybridization ratios (black squares), and the calculated behavior from our model (black curve), for the AON1 (left) and AON2 (right) sequences. In the model, the variables are set to T = 37 °C, [Na^+^] = 0.025 M and J_H_ = 4.31 kcal/mol, J_S_ = 5.5 cal/mol for AON1, and J_H_ = 2.62 kcal/mol and J_S_ = 1.93 cal/mol for AON2

In Fig. 3, we present the application of our theoretical model to the binding of the AON to the Target sequence. The predicted data as represented by the black curve is the result of direct calculation using our model, and is compared to our experimental results from Fig. 2.

Most noticeably, we observe that the appearance of the center peak is reliably reproduced in our model, in particular for the longer AON1. The coupling factors are, determined by fitting, J_H_ = 4.31 kcal/mol and J_S_ = 5.5 cal/mol for AON1, and J_H_ = 2.62 kcal/mol and J_S_ = 1.93 cal/mol for AON2, making them corrections significantly smaller than the binding energies per base pair [10]. The behavior closer to the edges is less exactly reproduced by our calculations, in particular when the mismatch is close to the 5′-end. This inaccuracy could be the result of our model not explicitly taking cases where only parts of the DNA strands hybridize into account. This effect that would be more pronounced when the mismatch is close to either end, in particular if it affects the stabilization of the silver cluster, which occurs on the 5′-end.

## Discussion

In this work, we present a new method of measuring DNA–DNA hybridization, and use it to determine the sensitivity of potential AON sequences to single-nucleotide mismatches. The effect of a base mismatch has historically been suggested to be independent of location [31], in contradiction to more recent experimental work [13], and our experimental findings in this article. In our measurements, we observe a strong lack of sensitivity to mismatches close to the center of the DNA strand, as well as close to the edges. This behavior around the center in particular can be reproduced through the application of our theoretical model.

In our modeling of the system, we approximate the behavior by initially treating the two halves on either side of the mismatch as separate pairs of DNA strands. Interactions between the strands are introduced through linear coupling terms, J_H_ and J_S_. These terms introduce a small negative contribution to the binding affinity. Of course, local interactions close to the mismatch can lead to various conformations, making it likely that this approximation is only applicable when J_H_ ≪ ΔH, and J_S_ ≪ ΔS. As the coupling factors are well over an order of magnitude smaller than the binding energies of the strands, the approximation can be considered appropriate.

The center peak in the mismatch dependency, that is most prominent for AON1, is reproduced by our model. This implies that for strands of this length, the two halves can be long enough to expect them to form hybrids by themselves. Considering the length of the AON1, 23 bases, this is not unreasonable, as 10–11 basepairs typically correspond to melting temperatures well above 37 °C [32]. The length of the AON represents a distinct difference between AON1 and AON2. The shorter, 20 base AON2 can not be represented by two halves of 10 basepairs or more in length, and therefore both halves can not be considered completely thermodynamically stable. This expected distinction between the two AONs is indeed shown by our model, and by our experimental results, where AON2 does not show significantly increased binding for mismatches close to the center. As a result, we conclude that the average sensitivity to a mismatch depends strongly on the length of the AON in this range.

The probe method we introduce allows the experiments to be performed in free solution, in conditions close to physiological, and we suggest they represent the behavior in living cells more closely than previous work on surface-bound DNA [13]. Therefore, this technique is of interest for the development of AON-based treatments for genetic diseases such as DMD. In our measurements, the binding efficiencies of AON1 do not dip below 55%, making the sequence generally fairly insensitive to mismatches. In the context of the strand’s functionality as an AON, this raises questions about the effectiveness of strands of this length. Under these conditions, the AON can be considered to lack the specific targeting as is required for patient treatment, implying that use of a shorter strand such as AON2, or one of a different sequence may be needed.

It must be noted, however, that while our conditions come close to physiological ones in temperature (37 °C), and pH (7.4), other effects have to be considered. Most notably, in treatments, the target exon and AON consist of (modified) RNA strands, not DNA, as used in our experiments. The different molecular and helical structure of RNA could cause it to have an altered mismatch dependency as well, most notably for some of the more constrained AON modifications such as locked nucleic acids (LNA) [35]. Additionally, the DNA concentrations used in our experiments are likely to be higher than the RNA concentrations found inside cells. Furthermore, the relatively high concentrations of salts in cells can strongly effect DNA dynamics as well. Silver ions in particular are known to interact strongly with ssDNA [22, 36] and although the silver clusters are only synthesized after DNA–DNA hybridization, it is difficult to predict how these interactions may affect our results. Our new experimental method, however, seems generally applicable to other sequences and conditions, and justifies further exploration.

## Conclusions

The main result of this article is the introduction of a new method of measuring DNA–DNA hybridization, using the formation of DNA-stabilized fluorescent silver clusters. To accomplish this, we expressed DNA–DNA binding through a strong fluorescence, which can be accurately measured in bulk under conditions close to physiological. We used this method to determine the hybridization rates of two DNA oligonucleotides with a longer target strand, and its dependency on nucleotide mismatches. As a secondary result, we introduce a simple model that is consistent with our experimental results. In doing so, we gained insights in the effectiveness as therapeutic oligonucleotides and the likelihood of off-target binding for these two sequences. We observed that, in particular for our longer 23-base strand, the hybridization is significantly less sensitive to mismatches located close to the center of the strand. These findings are an important step for understanding the effect of mismatches on the hybridization of oligonucleotides. Our probe method can be a valuable tool in investigating these effects further, to contribute to the design of AON therapies in particular.

## Additional file

**Additional file 1: Table S1.** DNA sequences used in the form of synthetic oligonucleotides in the presented DNA-DNA hybridization experiments. The designation ‘MMx’ refers to a single nucleotide mismatch on the xth location from the 5’ end of the AON sequence.
