# Supplementary material for: Measuring DNA hybridization using fluorescent DNA-stabilized silver clusters to investigate mismatch effects on therapeutic oligonucleotides
Source: J Nanobiotechnology. 2018 Apr 6;16:37. doi: 10.1186/s12951-018-0361-2 (PMC5887185; doi:10.1186/s12951-018-0361-2)
Supplement: Supplementary file 1 — Additional file 1: Table S1. DNA sequences used in the form of synthetic oligonucleotides in the presented DNA-DNA hybridization experiments. The designation ‘MMx’ refers to a single nucleotide mismatch on the xth location from the 5’ end of the AON sequence. [file 12951_2018_361_MOESM1_ESM.pdf]

Name	DNA sequence	Name	DNA sequence
Probe-AON1	TGCCTTTTGGGGACGGATATAGTTCAATATTTTAGTGTCTCC	Probe-AON2	TGCCTTTTGGGGACGGATAGGTCACCCACCATCACCCTC
Probe-AON1-MM1	TGCCTTTTGGGGACGGATAAAGTTCAATATTTTAGTGTCTCC	Probe-AON2-MM1	TGCCTTTTGGGGACGGATATGTCACCCACCATCACCCTC
Probe-AON1-MM2	TGCCTTTTGGGGACGGATATTGTTCAATATTTTAGTGTCTCC	Probe-AON2-MM2	TGCCTTTTGGGGACGGATAGTTCACCCACCATCACCCTC
Probe-AON1-MM3	TGCCTTTTGGGGACGGATATATTTCAATATTTTAGTGTCTCC	Probe-AON2-MM3	TGCCTTTTGGGGACGGATAGGACACCCACCATCACCCTC
Probe-AON1-MM4	TGCCTTTTGGGGACGGATATAGATCAATATTTTAGTGTCTCC	Probe-AON2-MM4	TGCCTTTTGGGGACGGATAGGTTACCCACCATCACCCTC
Probe-AON1-MM5	TGCCTTTTGGGGACGGATATAGTACAATATTTTAGTGTCTCC	Probe-AON2-MM5	TGCCTTTTGGGGACGGATAGGTCTCCCACCATCACCCTC
Probe-AON1-MM6	TGCCTTTTGGGGACGGATATAGTTTAATATTTTAGTGTCTCC	Probe-AON2-MM6	TGCCTTTTGGGGACGGATAGGTCATCCACCATCACCCTC
Probe-AON1-MM7	TGCCTTTTGGGGACGGATATAGTTCTATATTTTAGTGTCTCC	Probe-AON2-MM7	TGCCTTTTGGGGACGGATAGGTCACTCACCATCACCCTC
Probe-AON1-MM8	TGCCTTTTGGGGACGGATATAGTTCATTATTTTAGTGTCTCC	Probe-AON2-MM8	TGCCTTTTGGGGACGGATAGGTCACCTACCATCACCCTC
Probe-AON1-MM9	TGCCTTTTGGGGACGGATATAGTTCAAAATTTTAGTGTCTCC	Probe-AON2-MM9	TGCCTTTTGGGGACGGATAGGTCACCCTCCATCACCCTC
Probe-AON1-MM10	TGCCTTTTGGGGACGGATATAGTTCAATTTTTTAGTGTCTCC	Probe-AON2-MM10	TGCCTTTTGGGGACGGATAGGTCACCCATCATCACCCTC
Probe-AON1-MM11	TGCCTTTTGGGGACGGATATAGTTCAATAATTTAGTGTCTCC	Probe-AON2-MM11	TGCCTTTTGGGGACGGATAGGTCACCCACTATCACCCTC
Probe-AON1-MM12	TGCCTTTTGGGGACGGATATAGTTCAATATATTAGTGTCTCC	Probe-AON2-MM12	TGCCTTTTGGGGACGGATAGGTCACCCACCTTCACCCTC
Probe-AON1-MM13	TGCCTTTTGGGGACGGATATAGTTCAATATTATAGTGTCTCC	Probe-AON2-MM13	TGCCTTTTGGGGACGGATAGGTCACCCACCAACACCCTC
Probe-AON1-MM14	TGCCTTTTGGGGACGGATATAGTTCAATATTTAAGTGTCTCC	Probe-AON2-MM14	TGCCTTTTGGGGACGGATAGGTCACCCACCATTACCCTC
Probe-AON1-MM15	TGCCTTTTGGGGACGGATATAGTTCAATATTTTTGTGTCTCC	Probe-AON2-MM15	TGCCTTTTGGGGACGGATAGGTCACCCACCATCTCCCTC
Probe-AON1-MM16	TGCCTTTTGGGGACGGATATAGTTCAATATTTTATTGTCTCC	Probe-AON2-MM16	TGCCTTTTGGGGACGGATAGGTCACCCACCATCATCCTC
Probe-AON1-MM17	TGCCTTTTGGGGACGGATATAGTTCAATATTTTAGAGTCTCC	Probe-AON2-MM17	TGCCTTTTGGGGACGGATAGGTCACCCACCATCACTCTC
Probe-AON1-MM18	TGCCTTTTGGGGACGGATATAGTTCAATATTTTAGTTTCTCC	Probe-AON2-MM18	TGCCTTTTGGGGACGGATAGGTCACCCACCATCACCTTC
Probe-AON1-MM19	TGCCTTTTGGGGACGGATATAGTTCAATATTTTAGTGACTCC	Probe-AON2-MM19	TGCCTTTTGGGGACGGATAGGTCACCCACCATCACCCAC
Probe-AON1-MM20	TGCCTTTTGGGGACGGATATAGTTCAATATTTTAGTGTTTCC	Probe-AON2-MM20	TGCCTTTTGGGGACGGATAGGTCACCCACCATCACCCTT
Probe-AON1-MM21	TGCCTTTTGGGGACGGATATAGTTCAATATTTTAGTGTCACC		
Probe-AON1-MM22	TGCCTTTTGGGGACGGATATAGTTCAATATTTTAGTGTCTTC		
Probe-AON1-MM23	TGCCTTTTGGGGACGGATATAGTTCAATATTTTAGTGTCTCT		
19b-Probe	TGCCTTTTGGGGACGGATATA		
Target Sequence	TGGCTTTCTCTGCTTGATCAAGTTATAAAATCACAGAGGGTGATGGTGGGTGACCTTGAGGATATCAACGAGATGATCATCAAGCAGAAG		

Table S1. DNA sequences used in the form of synthetic oligonucleotides in the presented DNA-DNA hybridization experiments. The designation 'MMx' refers to a single nucleotide mismatch on the xth location from the 5' end of the AON sequence.
